# Prognostic Significance of Survivin Expression in Patients with Ovarian Carcinoma: A Meta-Analysis

**DOI:** 10.3390/jcm10040879

**Published:** 2021-02-21

**Authors:** Beata Gąsowska-Bajger, Agnieszka Gąsowska-Bodnar, Paweł Knapp, Lubomir Bodnar

**Affiliations:** 1 Institute of Chemistry, Opole University, 45-052 Opole, Poland; 2 Department of Gynecology and Gynecologic Oncology, Warmia and Masuria Oncology Centre of The Ministry of The Interior and Administration’s Hospital, 10-228 Olsztyn, Poland; agnga@mp.pl; 3 Department of Gynecology and Gynecologic Oncology, Medical University of Białystok, 15-276 Białystok, Poland; knapp@umb.edu.pl; 4 Department of Oncology and Immuno-Oncology, Warmia and Masuria Oncology Centre of The Ministry of The Interior and Administration’s Hospital, 10-228 Olsztyn, Poland; lubo@esculap.pl; 5 Department of Oncology, University of Warmia and Masuria,10-228 Olsztyn, Poland

**Keywords:** ovarian cancer, survivin, meta-analysis, prognostic factor

## Abstract

Background: Survivin belongs to the protein family of inhibitors of apoptosis (IAP) and is a regulator of the cell cycle and apoptosis. The aim of this study was to assess the clinical and prognostic significance of expression survivin in patients with ovarian cancer. Methods: We systematically searched for articles in PubMed, the American Chemical Society (Publications), Medline, the Royal Society of Chemistry, Scopus and the Web of Science. Patient clinical data, overall survival (OS), disease-free survival (DFS), and survivin expression were extracted from individual studies. We performed statistical analysis using the STATA 16 package. Eighteen publications containing data from 2233 patients with ovarian cancer were included in this meta-analysis. Results: We found an adverse effect of survivin expression on OS (risk ratio (HR): 1.60; 95% confidence interval (CI): 1.33–1.93, *p* = 0.00) but this was not observed on DFS (HR: 1.06; 95% CI: 0.55–2.05, *p* = 0.87). The analysis of clinicopathological parameters showed that survivin expression was associated with the histological grades (G1–2 vs. G3) (odds ratio (OR) = 0.53, 95% CI: 0.34–0.83, *p* = 0.01) and: International Federation Gynecology and Obstetrics (FIGO) stage (I–II vs. III–IV) (OR = 0.22, 95% CI: 0.09–0.55, *p* = 0.00), but it was not significantly correlated with the histological subtype (OR = 1.14, 95% CI: 0.83–1.58, *p* = 0.42). Conclusions: Our meta-analysis suggests that survivin expression may be a marker of poor prognosis in ovarian cancer. Survivin expression was associated with parameters of greater aggressiveness of ovarian cancer. Prospective studies are needed to confirm our results indicating that survivin expression can be used as an ovarian cancer biomarker.

## 1. Introduction

Ovarian carcinoma is the female reproductive system cancer with the worst prognosis [1,2] after cervical and uterine cancer [3], and accounts for 1.9% of all cancer deaths [4]. Ovarian cancer is more common in highly developed countries, and the number of new cases diagnosed is steadily increasing [4]. According to the GLOBCAN report, in 2018, 294,414 women in the world had ovarian cancer, which accounted for 1.6% of all cancers, while 184,799 deaths due to this cancer were reported [4]. Most cases of ovarian cancer occur in women between 50 and 70 years old. Risk factors for ovarian cancer include, but are not limited to, genetic alterations, fewer pregnancies, late menopause, endometriosis, polycystic ovary syndrome, and pelvic inflammatory disease [5]. The late diagnosis is caused by the lack of characteristic clinical symptoms and the lack of sensitive and specific tests that can detect it at an early stage of development [6,7,8]. Despite significant advances in the treatment of ovarian cancer, the overall 5-year survival regardless of advanced disease is about 30% [9]. Therefore, there is a need to look for new biomarkers that could indicate groups of patients with ovarian cancer with a poor prognosis for the more effective management of the therapeutic process.

Survivin, discovered in 1997 by Ambrosini and co-workers [10], plays a key role in the process of programmed cell death (apoptosis) and the regulation of cell division [11]. It is the smallest protein in the IAP mammal family, consisting of 142 amino acids, and its molecular weight is about 16.5 kDa. Human survivin protein is encoded by the BIRC5 gene located on 17q25, containing 14.7 kb and consisting of four exons and three introns. Survivin is expressed during embryonic development [11] and is commonly detected in foetal tissues but is not observed in normal adult tissues [10]. Survivin is present in both the cytoplasm and the cell nucleus [12,13]. Increased survivin expression is associated with a worse prognosis in various cancers, e.g., lung [14], colon [15], bladder [16] and prostate cancer [17], reduced apoptosis, worse response to treatment (chemotherapy) and shortened survival in patients [18,19]. Previous retrospective analyses of individual studies assessing the role of survivin expression in patients with ovarian cancer show conflicting results. To obtain a more accurate assessment of the clinical–pathological value and prognostic expression of survivin in ovarian cancer, we conducted a systematic literature review and meta-analysis of the available studies.

The primary endpoint of our meta-analysis was to assess the prognostic value of survival expression, i.e., the effect on overall survival and disease-free survival/relapse (DFS/RFS) among patients with ovarian cancer. The secondary endpoint was the assessment of the effect of survivin expression on the parameters of disease aggressiveness according to the International Federation Gynecology and Obstetrics (FIGO) stage, histological subtype, and histological grade.

## 2. Material and Methods

### 2.1. Search Strategy and Study Selection

A systematic literature search was performed during the period January 2000–April 2019 in the electronic databases the American Chemical Society (Publications), Medline, PubMed, the Royal Society of Chemistry, Scopus and the Web of Science. The searches were conducted using combinations of the following words: “survivin and ovarian”, “cancer”, “survivin expression and ovarian cancer”, “survivin expression”, “survivin”, “ovarian cancer” and “expression”. We focused only on articles in which research was carried out in humans.

We adopted the following inclusion criteria for our systematic review: (i) articles published in English in the form of full-text publications; (ii) studies performed on tissues from human subjects; (iii) survivin expression assessed by immunohistochemistry (IHC) or polymerase chain reaction (PCR) or reverse transcription polymerase chain reaction (RT-PCR): (iv) the relationship between survivin expression and the effect on the overall survival and/or disease-free survival were shown; and (v) the authors presented the sample size, hazard ratios (HRs) and their 95% confidence intervals (CIs) or other information that could help to deduce the data of survival function analysis.

When subsequent articles were published by the same research groups, the most recently published or most relevant single article was selected to prevent the duplication of data from the same patients. We did not attempt to use other search restrictions according to more specific methodological features. Therefore, the following exclusion criteria were used: (i) studies published as conference reports or review papers or letters to the editor; (ii) studies performed only on ovarian cancer cell lines and/or on animal models; and (iii) studies that did not provide sufficient data from which to obtain odds ratios (ORs), HRs and its 95% CIs.

### 2.2. Data Extraction

To minimise bias, two independent reviewers (B.G.B. and L.B.) assessed the studies based on abstracts during the search. If the study seemed appropriate, the entire manuscript was then evaluated and the study was considered “appropriate” if it met the inclusion criteria and did not meet the exclusion criteria. When analysing the publication, the completeness of the following data was noted: first author’s name, year of publication, country, number of patients, clinical and pathological features, HRs with 95% CIs regarding OS and DFS.

### 2.3. Quality Assessment

The Newcastle–Ottawa (NOS) quality rating scale was used to assess the quality of the original studies. The quality of selection, comparability and the exposure or performance of the study participants were the three main parameters. Studies with scores from 0 to 3, 4 to 6, and 7 to 9 were considered low, moderate, and high, respectively. Scores greater than 6 were considered high quality and included in our meta-analysis. A higher score means better methodological quality. Data extraction and quality assessment for each included study were performed independently by the three authors, and misunderstandings were resolved by consensus.

### 2.4. Statistical Analysis

The obtained data were analysed using the STATA 16 package. Pooled HR and odds ratio (OR) with 95% CI were used to present associations between survivin expression with ovarian cancer prognosis (DFS and OS) and clinicopathological factors, respectively. If variables’ HRs with their 95% CIs for overall survival (OS) or disease-free survival (DFS) were reported in the text, they were extracted directly; otherwise, data were calculated according to the method provided by Tierney [20].

Heterogeneity between studies was examined using the Chi-square-based Q test, in which I^2^ indicates the level of heterogeneity. I^2^ < 50% or *p*-heterogeneity > 0.1 represents low heterogeneity; in this case, a fixed effects model was used, otherwise a random effects model was selected. The probability value, *p* < 0.05 was considered statistically significant.

## 3. Results

### 3.1. Search Results

A total of 800 articles were identified from the primary literature search in the electronic databases PubMed, American Chemical Society (ACS, Scopus and Medline). After excluding duplicate citations, review articles, letters and studies which did not meet the inclusion criteria, twelve publications were included in the meta-analysis.

After the careful analysis of all 539 articles found in the database, some were removed due to the fact that they were duplicated. The remaining 261 articles were further reviewed and analysed. Then, 240 of them were excluded because they were review articles, article comments and conference materials that did not contain the data required for this meta-analysis. Twenty-one articles were left after exclusion. Then, three more articles were excluded: two articles were duplicates of research and one article was a commentary. Finally, 18 studies published between 2003 and 2016 were included in the meta-analysis.

**Figure 1:**
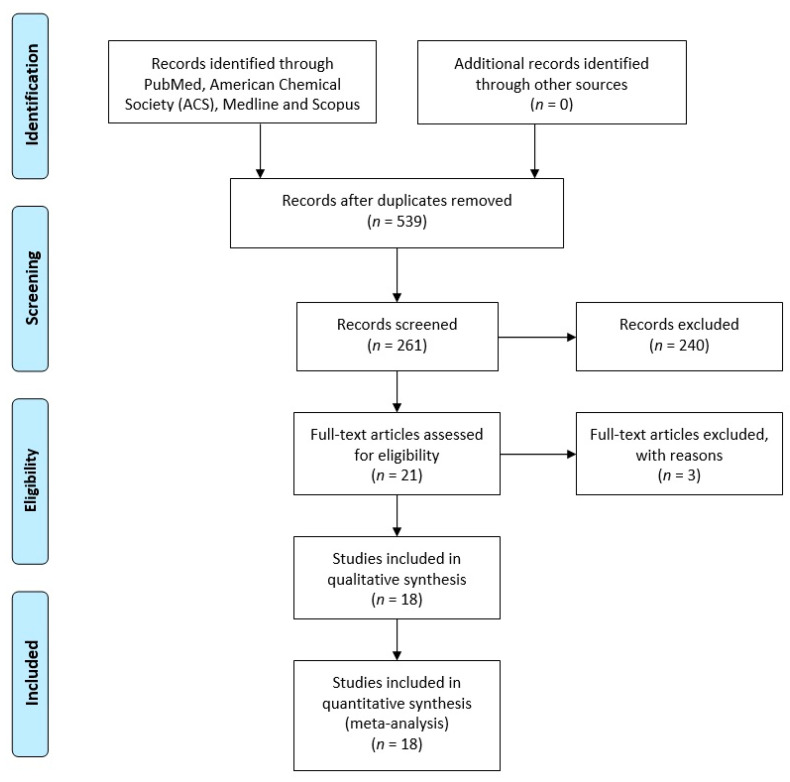
PRISMA (Preferred Reporting Items for Systematic Reviews and Meta-Analyses) 2009 flow diagram [30]. Selection of studies.

Overall, 18 articles were included in the meta-analysis to evaluate the prognosis and the clinical significance of survivin in ovarian cancer, whereas 10 [18,21,22,23,24,25,26,27,28,29] reported data for HR with 95% CI (directly and indirectly) (Figure 1) [30].

### 3.2. Basic Information for Inclusion in the Literature

As a result, 18 studies published between 2003 and 2016 met the accepted inclusion conditions for this meta-analysis, including a total 2233 patients with survivin expression data and ovarian cancer. The basic characteristic descriptions of the 18 studies are summarised in Table 1. Overall, four studies were included from Turkey [26,28,31,32], three from China [7,33,34], five from Poland [25,27,29,35,36], two from the USA [21,37], and one each from Greece [24], Norway [23], Japan [38] and Italy [22]. Survivin expression was measured by IHC (*n* = 17) and RT-PCR (*n* = 1).

### 3.3. Survivin Expression Assessment in Ovarian Benign Tumour, Borderline Ovarian Tumour and Ovarian Carcinoma

A total of 18 studies [7,21,22,23,24,25,26,27,28,29,31,32,33,34,35,36,37,38] reported survivin expression in ovarian cancer, including 1540 (69%) cases of the total 2233 ovarian cancer patients. In six publications used in this meta-analysis, the authors divided all cases presenting survivin expression into three groups: ovarian benign tumour, borderline ovarian tumour and ovarian carcinoma. The analysis showed that the highest expression of survivin was found in the ovarian carcinoma group, in 78.0% (1448/1856) patients, with a lower expression found in the borderline group (45.7%; 59/129), and the lowest in the benign group (21.7%; 46/212). There was no expression of survivin in the normal ovarian tissue (0%; 0/56).

**Figure 2:**
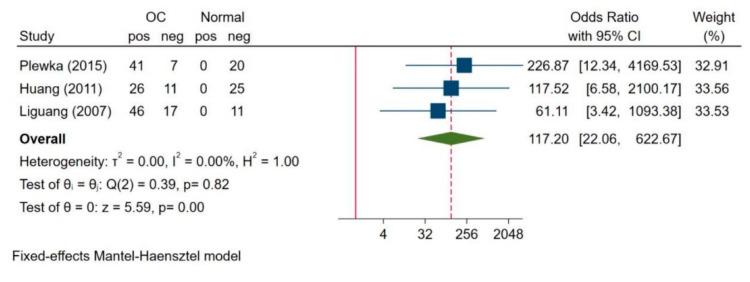
Comparison of survivin expression between ovarian carcinoma and ovarian normal tissue [33,36,37]. Abbreviations: OC = Ovarian Cancer.

In three studies reporting survivin expression in ovarian carcinoma and normal ovarian tissue, we did not observe any heterogeneity between the two groups (I^2^ = 0.00%, H^2^ = 1.0, *p* = 0.82); therefore, we used the fixed-effects model for analysis (Figure 2). We found that survivin expression was significantly higher in ovarian carcinoma than in normal ovarian tissue (OR = 117.20; 95% CI: 22.06–622.67, *p* = 0.00).

**Figure 3:**
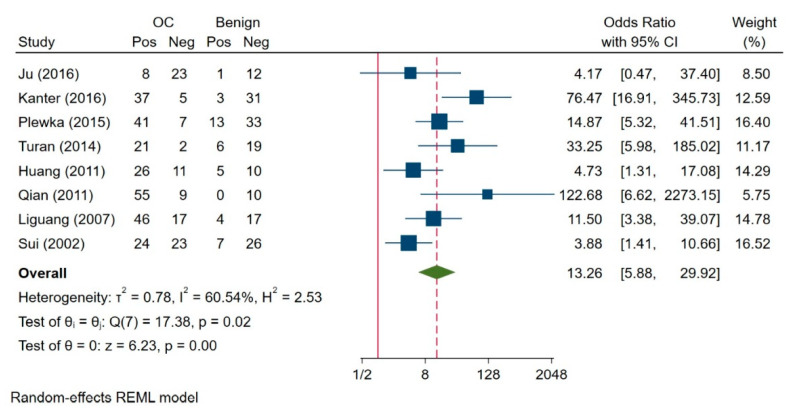
Comparison of survivin expression between ovarian carcinoma and benign tumour [3,7,31,32,33,36,38]. Abbreviations: OC =Ovarian Cancer; REML = The Restricted Maximum Likelihood.

Heterogeneity was observed between the groups of ovarian carcinoma and benign tumour patients (I^2^ = 60.54%, H^2^ = 2.53, *p* = 0.02) in eight studies; therefore, we used a random-effects model for analysis (Figure 3). We found that survivin expression was significantly higher in ovarian carcinoma compared to benign ovarian tumours (OR = 13.26; 95% CI: 5.88–29.92, *p* = 0.00).

**Figure 4:**
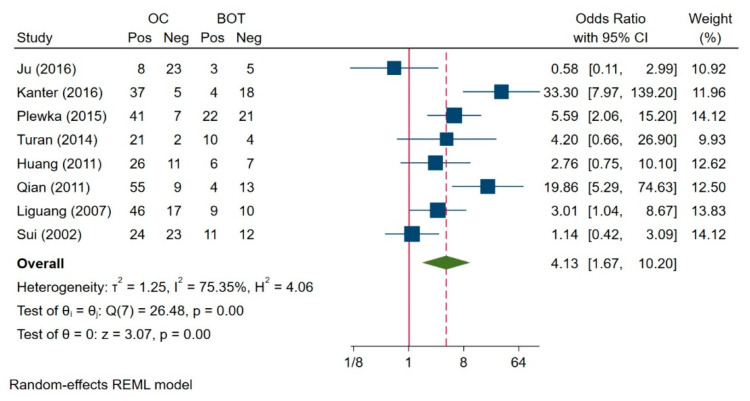
Comparison of survivin expression between ovarian carcinoma and borderline ovarian tumour [3,7,31,32,33,36,38]. Abbreviations: OC = Ovarian Cancer; BOT = Borderline Ovarian Tumour; REML = The Restricted Maximum Likelihood.

We found heterogeneity between the groups of ovarian carcinoma and borderline ovarian tumour patients (I^2^ = 75.35%, H^2^ = 4.06, *p* = 0.00) in eight included studies; therefore, the random-effects model was used for this analysis (Figure 4). The survivin expression in ovarian carcinoma was found to be significantly higher than in the borderline ovarian tumour patients (OR = 4.13, 95% CI: 1.67–10.20, *p* = 0.00).

### 3.4. Survivin Expression and FIGO Stage

**Figure 5:**
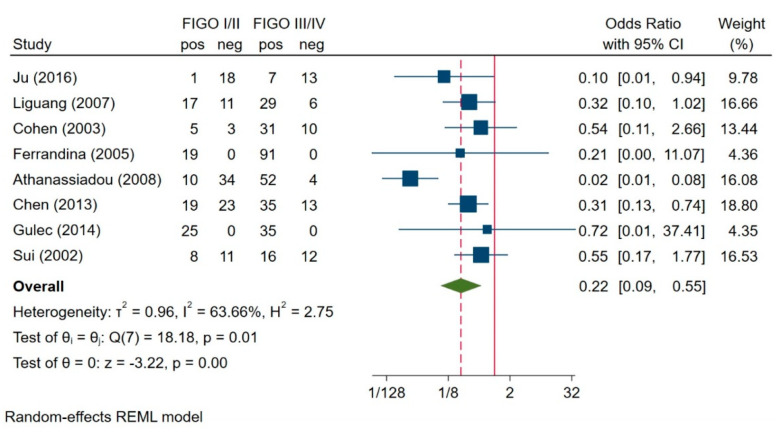
Association between survivin expression and FIGO stage [7,21,22,24,28,34,37,38]. Abbreviations: REML = The Restricted Maximum Likelihood.

A total of seven studies which evaluated the correlation of survivin expression with the stage were included [7,21,22,24,28,34,37,38]. Of the 204 patients in stages I and II, 104 (50.98%) had positive survivin expression, and 296 (83.62%) of the 354 patients in stage III and IV had positive survivin expression. The test of heterogeneity for these seven studies was significant (I^2^ = 63.66%, H^2^ = 2.75, *p* = 0.01); therefore, we used a random-effects model for analysis. The results of the meta-analysis (Figure 5) showed that patients in FIGO stages I and II had a significantly lower expression of survivin than those in FIGO stages III and IV (OR = 0.22, 95% CI: 0.09–0.55, *p* = 0.00).

### 3.5. Association between Survivin Expression and Histological Grade

**Figure 6:**
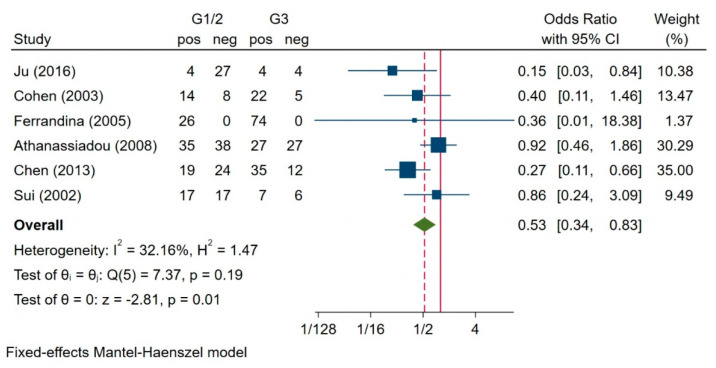
Association between survivin expression and histologic grade [7,21,22,24,34,38].

The meta-analysis was performed on six studies investigating the association between the survivin expression with the histological tumour grade [7,21,22,24,28,34,37,38]. Of the 229 patients in grades 1 and 2, 116 (50.22%) had enhanced survivin expression, and 169 (75.78%) of the 223 patients in grade 3 had enhanced survivin expression. Heterogeneity was not significant between the two groups; therefore, the fixed-effects model was used for this analysis (I^2^ = 32.16%, H^2^ = 1.47, *p* = 0.19) (Figure 6). We found that patients with grades 1 and 2 had a significantly lower expression of survivin than those with grade 3 (OR = 0.53; 95% CI: 0.34–0.83; *p* = 0.01).

### 3.6. Association between Survivin Expression and Histological Subtype

**Figure 7:**
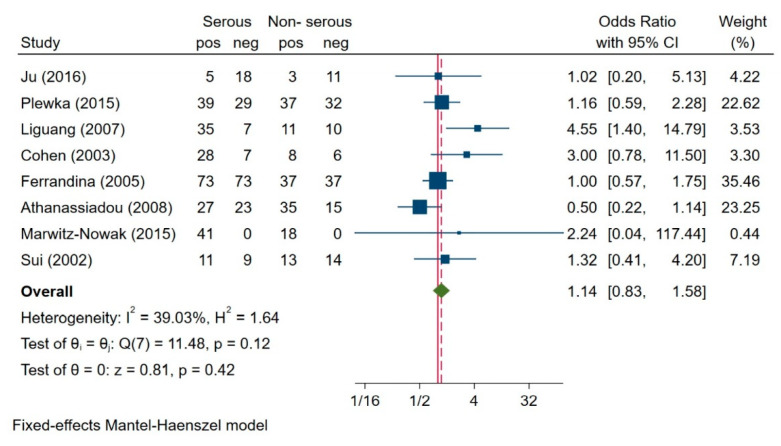
Association between survivin expression and histologic subtype [7,21,22,24,35,36,37].

From the studies qualified for the meta-analysis, we found that eight works assessed the role of survivin expression in different subtypes of ovarian cancer. Of the 151 patients with endometrioid cancer, 47 (27.5%) had enhanced survivin expression; of the 147 patients with the mucinous subtype, 82 (55.8%) had enhanced survivin expression; of the 332 patients with the serous subtype, 237 (71.4%) had enhanced survivin expression; of the seven patients with a poorly differentiated tumour, five (71.4%) had enhanced survivin expression; of the 16 patients with an undifferentiated tumour, 11 (67%) had enhanced survivin expression; and of the 16 patients with clear cell carcinoma, seven (43.75%) had enhanced survivin expression. The test of heterogeneity was not significant for eight studies (I^2^ = 39.03%, H^2^ = 1.64, *p* = 0.42); therefore, we used the fixed-effects model to compare the survivin expression between the serous and non-serous subtypes of ovarian carcinoma. Based on this meta-analysis, we found that patients with serous and non-serous subtypes of ovarian carcinoma showed no significant differences in survivin expression (OR = 1.14, 95% CI: 0.83–1.58, *p* < 0.48) (Figure 7).

### 3.7. Impact of Survivin Expression on Prognosis in Ovarian Cancer

**Figure 8:**
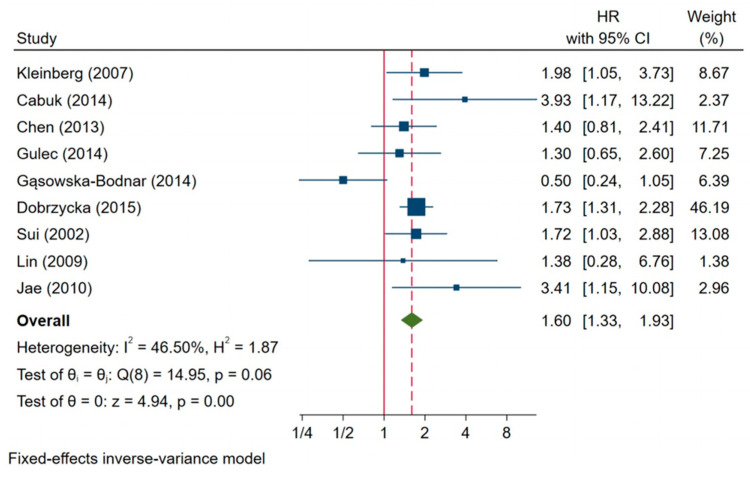
Impact of survivin expression on overall survival [3,23,26,27,28,29,34,38].

In this meta-analysis, we found nine studies presenting a correlation between survivin expression and OS, including a total of 954 patients with ovarian carcinoma. We applied Kaplan–Meier and Tierney methods to calculate HR. Heterogeneity was not demonstrated in the subgroup analysis (I^2^ = 46.50%, H^2^ = 1.87, *p* = 0.06). The results of the meta-analysis of a fixed effect model showed that the combined HR was 1.60, with a 95% CI of 1.33–1.93 and *p* = 0.00. The survivin expression was associated with a 60% higher risk of death than the no expression of this protein in patients with ovarian cancer. Importantly, the subgroup analysis revealed no heterogeneity (Figure 8).

**Figure 9:**
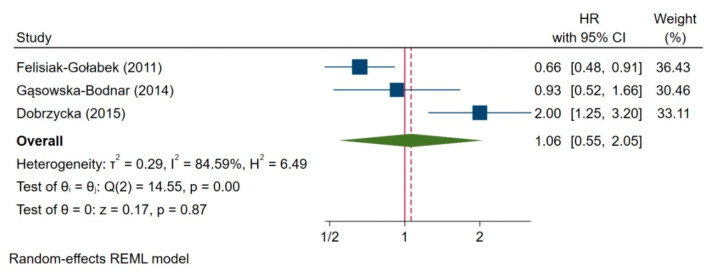
Impact of survivin expression on disease-free survival [25,27,29].

In addition, three studies were included where the disease-free survival data were presented for survivin expression in patients with ovarian carcinoma. The test of heterogeneity for those studies was significant (I^2^ = 84.59%, H^2^ = 6.49, *p* = 0.00), so the random-effects model was used for analysis. The pooled HR was 1.06 (95%CI: 0.55–2.05, *p* = 0.87), which indicated no significant effect of survivin expression on disease-free survival (Figure 9).

### 3.8. Sensitivity Analysis

We attached the sensitivity analysis to the manuscript. We conducted a sensitivity analysis to assess the impact of a specific publication on the overall estimate. The sensitivity analysis revealed that the elimination of a single one significantly changed the OR effect in the meta-analysis. After excluding one of the studies [27], the heterogeneity decreased dramatically in the overall survival analysis (I^2^ = 0%). In the case of the disease-free survival/recurrent-free survival analysis, with the exclusion of one study [29], the heterogeneity decreased to I^2^ = 2.81%. It follows that these studies have a significant impact on the collective risk assessment (Table 2).

### 3.9. Publication Bias

**Figure 10:**
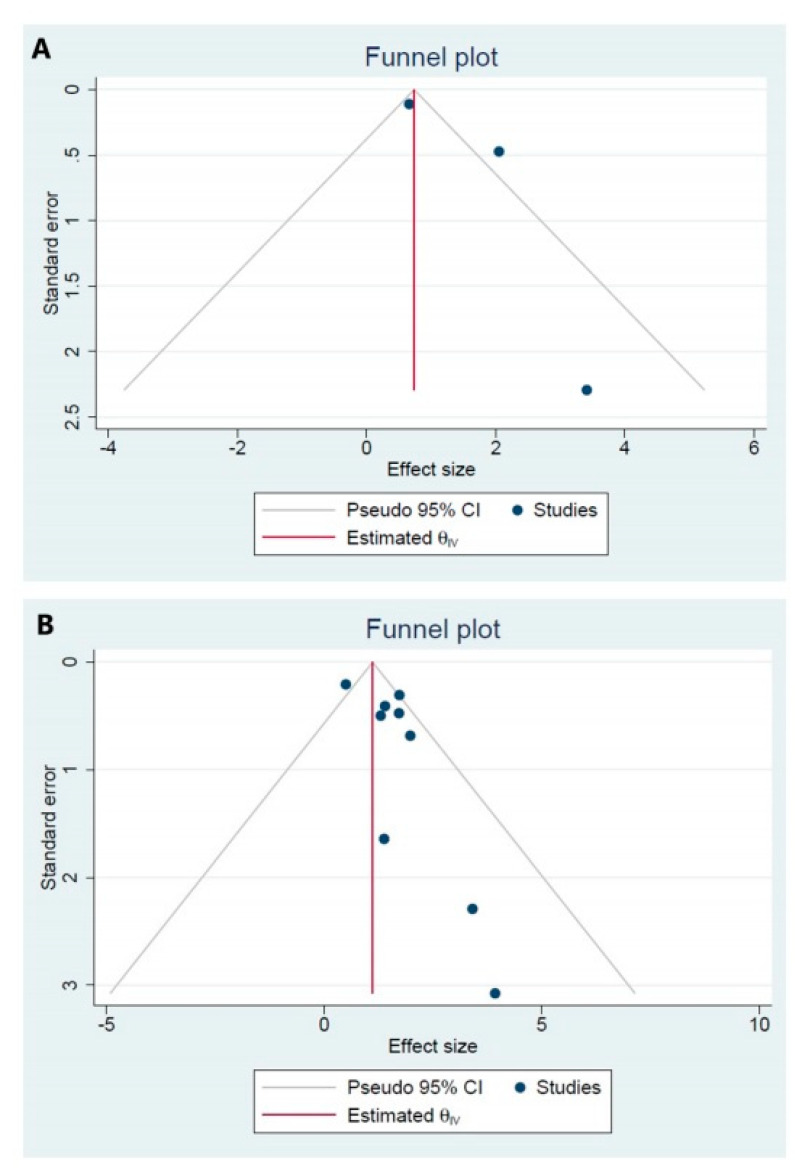
(**A**) Funnel plot for the estimation of potential publication bias. Begg’s funnel plot for disease-free survival; and (**B**) funnel plot for the estimation of potential publication bias. Begg’s funnel plot for overall survival.

We used Begg’s test to estimate publication bias in the our meta-analysis. We did not observe for publication in the Begg’s test between survivin expression and OS, and DFS in patients with ovarian carcinoma (Figure 10A,B).

## 4. Discussion

The purpose of this meta-analysis was to determine the clinical and prognostic significance of survivin in patients with ovarian cancer. We systematically summarised the existing evidence, performing a meta-analysis on a total of 18 studies. Our results indicated that higher survivin expression was associated with shorter overall survival in patients with ovarian cancer. We also found that survivin expression was associated with a higher tumour histological grade and advanced ovarian cancer according to FIGO, but the number of studies was limited.

Ovarian cancer is characterised by the worst prognosis of all gynaecological cancers. In 2019, The American Cancer Society estimated that around 21,750 women would get ovarian cancer and as many as 13,940 women would die of ovarian cancer in the United States [39].

Apoptosis, a programmed cell death process, is important in both carcinogenesis and cancer treatment. It can run on two main pathways: external and internal. The first is initiated by the ligation of death receptors of the tumour necrosis factor receptor family (TNR-F). The second is mainly induced by stress factors such as drugs or irradiation. Interest in survivin results from experimental studies that have shown that survivin can inhibit both external and internal apoptosis pathways [40]. Survivin is detected in embryonic and foetal development [11], while it is rarely seen in healthy adult tissues, except placental tissue, bone marrow stem cells and testicular tissue [41], but it can be reactivated during the onset of most cancers, including breast [42] stomach [43,44,45], lung [14,46] colorectal [47], oral [48] and ovarian [49] etc. Furthermore, strong survivin expression has been demonstrated in the pancreas, esophagus, endometrium, uterine cervix, ovary, melanoma and non-melanoma skin cancers, and neuroblastoma. The percentage of survivin-positive cases varies from 35% in gastric cancer to 93% in primary and metastatic malignant melanoma, with expression in 51–86% of ovarian cancers [21].

Survivin may inhibit apoptotic signalling induced by caspase-3, 7 and 9 and cytochrome c. It does not bind directly to caspases, but needs the XIAP protein (x-linked inhibitor of apoptosis protein 4). Survivin may also inhibit apoptosis in a caspase-independent manner by destabilising the mitochondrial apoptosis-inducing factor (AIF), which in turn causes DNA fragmentation [50]. In addition, survivin may affect other pathways associated with carcinogenesis such as the MAPK/ERK [51], Ras/STAT3 [52] pathways. The inhibition of this pathway by targeting ERK or MEK leads to the suppression of ovarian tumour growth. From the therapeutic perspective, PARP1 inhibition causes a loss of ERK2 stimulation by decreasing the activity of vascular endothelial growth factor (VEGF) and hypoxia inducible factor (HIF), and can be combined with survivin inhibitors [53]. It has also been observed that survivin overexpression may cause resistance to some cytostatics such as vincristine and cisplatin, paclitaxel and shorter disease-free survival and overall. Therefore, survivin may be a useful cancer biomarker and therapeutic target [49,54]. In addition, survivin may affect other pathways associated with carcinogenesis such as the MAPK/ERK [51], Ras/STAT3 [52] pathways.

Increased survivin expression is associated with a worse prognosis, reduced apoptosis, worse response to treatment and shortened survival in patients in various cancers. Survivin is commonly expressed in ovarian cancer and its expression levels are strongly associated with the proliferative activity of the tumours and the survival of the patients. It is possible that the prognostic impact of nuclear survivin expression could be particularly useful in the identification of patients who are at higher risk of ovarian carcinoma relapse.

In our meta-analysis, we compared the survivin expression between ovarian cancer and normal tissue, benign ovarian tumours, and borderline ovarian tumours. We found that survivin expression in ovarian cancer cells was statistically significantly higher than borderline ovarian tumours, benign ovarian tumours, and normal tissues. It is particularly important to observe that survivin expression was not observed in normal ovarian tissues, which may indicate that survivin is not activated and does not participate in the mechanisms of apoptosis in normal ovarian cells. Series of studies published by Huang [33], Kanter [32], Turan [31], Liguang [37] and colleagues showed that the expression of survivin in ovarian cancer was significantly higher than in borderline and benign tumours. A meta-analysis summarising other selected studies regarding survivin, including those published in Chinese and conducted mainly on the Asian population, also showed significantly higher survivin expression in ovarian cancer cells compared to normal ovarian tissue, benign ovarian tumours and borderline ovarian tumours [55].

In our meta-analysis, 17 studies evaluated the survivin expression by immunohistochemistry (IHC), and one by reverse-transcription polymerase chain reaction (RT-PCR); most studies used IHC to determine survivin expression because it has the advantage of showing exactly where the survivin is in the cell and allows the assays to remain reliable despite the passage of time, as most studies are conducted on retrospective archival material. In this study, we analysed the expression and clinical relevance of survivin in ovarian cancer. We found that survivin expression was positively correlated with the more advanced stages according to the FIGO classification system and the more aggressive histological grade G3. This suggests a relationship between advanced clinical stages, high-grade histology and survivin expression. In single studies assessing the relationship between survivin expression and clinical features of ovarian cancer, Altieri [11] found that survivin expression was associated with tumour metastasis. In turn, studies by Cohen [21], Ferrandina [22], Liguang [37] and Athanassiadou [24] and colleagues showed that the expression of survivin in ovarian cancer was closely related to the FIGO status, tumour grade and histological type, but showed no effect on the age of the patient. A meta-analysis conducted by He et al. showed a relationship between survivin expression and later clinical stage and lower histological differentiation, but the assessed studies mainly included the Asian population [55].

We found that higher survivin expression was associated with poorer overall survival, but not with DFS in patients with ovarian cancer. Analysing individual studies assessing the prognostic role of survivin, Cohen et al. [21] found that the expression of both nuclear and detectable survivin in the cytoplasm was not associated with total survival and DFS. Similarly, in the study of Ferandina et al. [22], no association between nuclear and cytoplasmic survivin expression with prognosis was observed. In turn, Chen et al. found that the cytoplasmic expression of survivin was an independent molecular prognostic marker in ovarian cancer [34]. A meta-analysis conducted by Li et al. on a group of 4600 patients from 10 studies and GEO (Gene Expression Omnibus) data showed that survivin expression was significantly associated with poorer overall survival in ovarian cancer. However, as indicated by the authors, the number of studies assessing non-Asian patients was small, which may be a limitation to this meta-analysis. In addition, the authors included data evaluating survivin expression reported using various methods in addition to immunohistochemical assessment [36].

Our meta-analysis is a comprehensive assessment of the clinical and prognostic significance of survivin in ovarian cancer, but several limitations should be noted. Firstly, in the studies included the criteria for determining positive or negative expression of survivin by IHC were inconsistent, which may affect the heterogeneity of the results obtained. Therefore, it is necessary to define a standard cut-off value. Secondly, when analysing the relationship between survivin expression and the clinical–pathological features of ovarian cancer, some studies lacked full data, which may cause heterogeneity and result in the decreased reliability of results.

## 5. Conclusions

In summary, despite the above-mentioned limitations, our meta-analysis suggested clinical–pathological and prognostic values of survivin expression in ovarian cancer. Current research shows that survivin expression may become a specific biomarker in patients with ovarian cancer and its occurrence indicates a poor prognosis.

## Figures and Tables

**Table 1:** Main characteristics of the included studies.

Autor	Reference	Country	Year	No. of P.	Mean Age (Range)	Method	Tumour Grade G1/G2/G3	FIGO Stage (I–II/III–IV)
Huang Ju	[33]	China	2016	60	-	IHC	-	-
Kanter M.	[32]	Turkey	2016	98	52 (18–87)	IHC	17/25 ^*^	15/27
Ju L.-L.	[7]	China	2016	60	48 (25–72)	IHC	-	19/20
Plewka D.	[36]	Poland	2015	137	45 (34–58)	IHC	-	-
Dobrzycka B.	[29]	Poland	2015	92	56 (32–76)	IHC	33 ^**^/59	21/71
Gąsowska-Bodnar A.	[27]	Poland	2014	66	60 (39–80)	IHC	-	-/60
Turan G.	[31]	Turkey	2014	62	48 (16–88) benign 39 (17–77) borderline 52 (36–82) malignant	IHC	-	6/17
Gulec U.K.	[28]	Turkey	2014	73	52.6 (17–78)	IHC	11 ^**^/47	41/15
Chen L.	[34]	China	2014	90	50 (22–75)	IHC,	22/21/47	42/48
Çabuk F.K.	[26]	Turkey	2014	60	54.5 (36–80)	IHC	8/21/31	25/35
Felisiak-Gołabek A.	[25]	Poland	2011	435	54.3 (20–78)	IHC	54/263/118	27/408
Nowak-Markwitz E.	[35]	Poland	2010	82	49 (49–75)	IHC	17/33/24	20/62
Athanassiadou P.	[24]	Greece	2008	100	62 (38–82)	IHC	34/39/27	44/56
Liguang Z.	[37]	China, USA	2007	114	56 (19–72)	RT-PCR	30 ^**^/33	28/35
Kleinberg L.	[23]	Norway	2006	220	63 (38–87) ^+^ 59 (25–81) ^++^	IHC	14/31/106	3/172
Ferrandina G.	[22]	Italy	2005	110	58.5 (25–84)	IHC	26 ^**^/74	19/91
Cohen C.	[21]	USA	2003	49	57 (29–76) ^•^ 63 (45–79) ^••^	IHC	7/15/27	5/41
Sui L.	[38]	Japan	2002	103	49 (16–77)	IHC	21/13/13	19/28

Abbreviations: IHC = immunohistochemistry; FIGO = International Fereration Gynecology and Obstetrics; * = G2+G3; ** = G1+G2; - = not applicable; + = primary diagnosis; ++ = disease recurrence; • = survivin negative; •• = survivin positive.

**Table 2:** Analysis sensitive for overall survival and disease-free survival/relapse (DFS/RFS).

Studies	References	HR	95% CI	Heterogeneity Test	
				I^2^ (%)	P
Overall Survival					
All studies (*n* = 9)		1.60	1.33–1.99	46.50	0.06
Omitting Kleinberg (2007)	[23]	1.57	1.29–1.91	51.66	0.04
Omitting Cabuk (2014)	[26]	1.57	1.30–1.89	45.30	0.08
Omitting Chen (2013)	[34]	1.63	1.34–1.99	52.35	0.04
Omitting Gulec (2014)	[28]	1.63	1.34–1.98	51.99	0.04
Omitting Gąsowska-Bodnar (2014)	[27]	1.73	1.43–2.10	0.00	0.69
Omitting Dobrzycka (2015)	[29]	1.50	1.16–1.93	51.34	0.04
Omitting Sui (2002)	[38]	1.58	1.30–1.93	52.92	0.04
Omitting Lin (2009)	[3]	1.60	1.33–1.94	53.08	0.04
Omitting Jae (2010)	[3]	1.56	1.29–1.89	46.27	0.07
Disease-Free Survival/ Recurrent-Free Survival					
All studies (*n* = 3)		1.06	0.55–2.05	84.59	0.00
Omitting Felisiak-Gołąbek (2011)	[25]	1.39	0.66–2.94	75.17	0.04
Omitting Gąsowska-Bodnar (2014)	[27]	1.13	0.38–3.36	93.12	0.00
Omitting Dobrzycka (2015)	[29]	0.72	0.54–0.96	2.81	0.31

## Data Availability

Data is contained within the article or supplementary material.
